# Macro-Micro Simulation for Polymer Crystallization in Couette Flow

**DOI:** 10.3390/polym9120699

**Published:** 2017-12-11

**Authors:** Chunlei Ruan, Kunfeng Liang, Enli Liu

**Affiliations:** 1 School of Mathematics and Statistics, Henan University of Science and Technology, Luoyang 471023, China; 13007559079@163.com; 2 College of Vehicle & Motive Power Engineering, Henan University of Science and Technology, Luoyang 471023, China; liangkunf@163.com

**Keywords:** macro-micro simulation, flow-induced crystallization, Monte Carlo method, crystal morphology

## Abstract

Polymer crystallization in manufacturing is a process where quiescent crystallization and flow-induced crystallization coexists, and heat/mass transfer on a macroscopic level interacts with crystal morphology evolution on a microscopic level. Previous numerical studies on polymer crystallization are mostly concentrated at a single scale; they only calculate macroscale parameters, e.g., temperature and relative crystallinity, or they only predict microstructure details, e.g., crystal morphology and mean size of crystals. The multi-scale numerical works that overcome these disadvantages are unfortunately based on quiescent crystallization, in which flow effects are neglected. The objective of this work is to build up a macro-micro model and a macro-micro algorithm to consider both the thermal and flow effects on the crystallization. Our macro-micro model couples two parts: mass and heat transfer of polymeric flow at the macroscopic level, and nucleation and growth of spherulites and shish-kebabs at the microscopic level. Our macro-micro algorithm is a hybrid finite volume/Monte Carlo method, in which the finite volume method is used at the macroscopic level to calculate the flow and temperature fields, while the Monte Carlo method is used at the microscopic level to capture the development of spherulites and shish-kebabs. The macro-micro model and the macro-micro algorithm are applied to simulate polymer crystallization in Couette flow. The effects of shear rate, shear time, and wall temperature on the crystal morphology and crystallization kinetics are also discussed.

## 1. Introduction

Semi-crystalline polymer products are currently widely used in industry [1]. During manufacture processing, polymer melts suffer from complex flow and thermal effects with the internal chains changing and folding to form different types of crystal structures. These microstructures are the key factors that determine the properties of the products [2]. Thus, it is very important to examine the evolution of crystal structures and the crystallization kinetics under different thermal and flow conditions.

**Figure 1:**
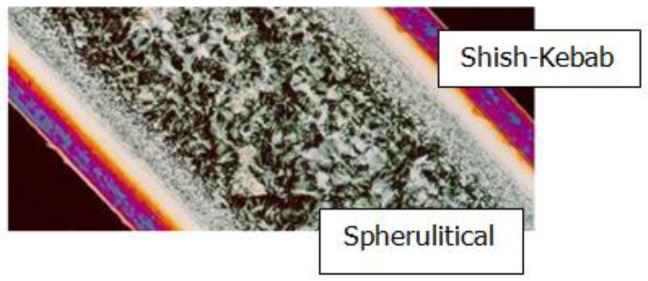
Spherulitic and shish-kebab structure in injection polymer product [3].

As reported in [2], the main types of crystal structures in injection polymer products are spherulitic structures and shish-kebab structures. Figure 1 shows the spherulitic and shish-kebab structures in injection polymer products [3]. This phenomenon is called the “skin-core-skin” structure. In fact, the shish-kebab structure appears highly ordered in the skin and the spherulitic structure appears in the core with essentially no preferred orientation. Generally, we assume that the spherulitic structure is thermally induced, while the shish-kebab structure is flow induced [2]. In other words, the spherulitic structure is caused by quiescent crystallization, while the shish-kebab structure is caused by flow-induced crystallization. Therefore, polymer crystallization in manufacturing is a process where quiescent crystallization and flow-induced crystallization coexist.

The lengths of the crystal structures are approximately 10^−3^–10^−5^ m (micro-scale), while the lengths of the products are approximately 10^0^–10^−2^ m (macro-scale) [4]. These structures are on different length scales, but still interact. For example, the heat/mass transfer at the macroscopic level changes the nucleation and growth rate of the crystals. Conversely, as the crystals develop, they release latent heat and change the viscosity of the melt. Hence, polymer crystallization is a typical multi-scale problem.

To date, many studies on the simulation of polymer crystallization have been reported, but most of them are only based on a single scale. For example, Goff et al. [5] used the finite difference method to solve the Nakamura crystallization kinetics equation and the energy equation to obtain the crystallization rate and temperature distribution in polymer products. Yang et al. [6] used the finite volume method to solve the enthalpy equation and obtained the distribution of temperature in the injection polymer product and gas-assisted injection polymer product. Although, the macroscopic works can accurately predict the crystallinity and temperature, they cannot reveal the details of the microstructure. Raabe [7] constructed a cellular automaton method to simulate the topology of spherulite growth. Liu et al. [8] presented a particle level set method to simulate polymer crystallization under isothermal and temperature gradient conditions. Wang et al. [9] constructed a phase field method to predict the growth of shish-kebabs. While the above microscopic works can predict the crystal morphology evolution, they cannot reflect the interaction between crystallization and thermal/flow conditions. Multi-scale methods can overcome these disadvantages. For example, Charbon et al. [4] presented a crystal front-tracking model and a heat transfer model for different scales, and used a coupled front-tracking technique with the finite difference method on different grids to capture crystal evolution at the micro-scale, and calculate temperature at the macro-scale. Ruan et al. [10,11] constructed a hybrid pixel coloring method with the finite volume method to capture the evolution of the crystal morphology and compute the crystalline kinetics and temperature in the polymer and short fiber reinforced system; Liu et al. [12] built up a level set method and finite volume method to simulate the polymer crystallization during the cooling stage. Spina et al. [13,14] developed a cellular automaton method and the finite element method to simulate the kinetics and topology of spherulite growth for polymer crystallization. However, it should be mentioned that the above multi-scale works are based on the quiescent crystallization where the flow effects have not been considered and the crystal structure is only the spherulite.

Couette flow is a typical shear flow that exists widely in experiments and manufacturing. Many numerical studies are based on Couette flow model. For example, Zinet et al. [15] used the Couette flow as an example to show the flow and thermal effects in non-isothermal polymer crystallization. Mu et al. [16] investigated the thermally and flow-induced crystallization behavior of semi-crystalline polymers in Couette flow. Rong et al. [17] explored the effects of flow-induced crystallization in Couette flow using multi-scale simulations.

In this paper, we build a macro-micro model and a macro-micro algorithm to simulate polymer crystallization in Couette flow. The macro-micro model contains two parts: mass and heat transfer of polymeric flow at the macroscopic level, and nucleation and growth of spherulites and shish-kebab structures at the microscopic level. The two suspensions model [18] is used to describe the system, where the Finite Extensible Non-linear Elastic dumbbell model with Peterlin’s approximation (FENE-P dumbbell model) is used to describe the amorphous phase and the rigid dumbbell model is used to describe the semi-crystalline phase. Equations for the nucleation and growth of spherulites and shish-kebabs are deduced using the Eder model [19]. The crystallization kinetics are calculated with the volume fraction. A coupled finite volume method using a Monte Carlo method is constructed, where the finite volume method is used at the macroscopic level to calculate the flow and temperature fields, and the Monte Carlo method is used at the microscopic level to capture the development of spherulites and shish-kebabs.

## 2. Macro-Micro Model

### 2.1. The Model for Heat and Mass Transfer of Polymeric Flow at the Macroscopic Level

**Figure 2:**
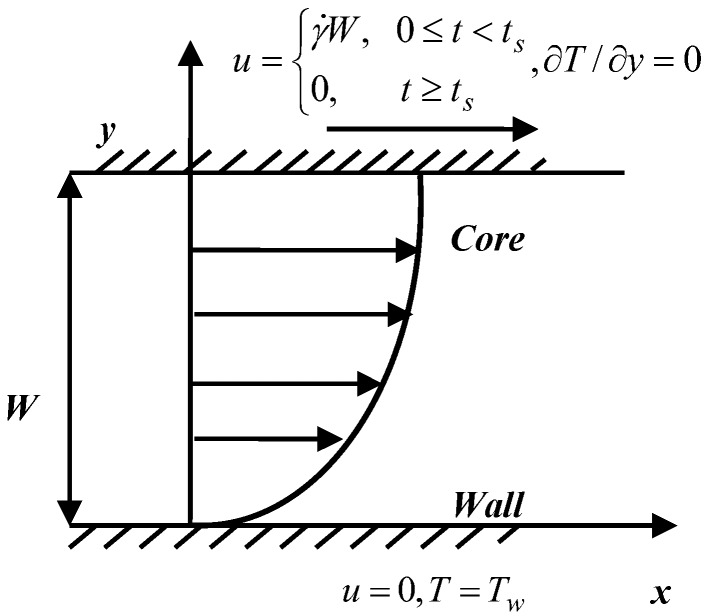
Geometry and boundary condition for Couette flow.

The governing equations for polymeric flow are the balance equations of mass, momentum and energy. For the Couette flow, polymer velocities automatically satisfy the incompressible condition. Therefore, the continuity equation will not be calculated. Considering the one-dimensional Couette flow shown in Figure 2, the momentum equation is:

(1) $$\rho \frac{\partial u}{\partial t}=\eta_{s}\frac{\partial^{2}u}{\partial y^{2}}+\frac{\partial \tau_{xy}}{\partial y}$$

and the energy equation is:

(2) $$\rho c_{p}\frac{\partial T}{\partial t}=k_{p}\frac{\partial^{2}T}{\partial y^{2}}+\rho \Delta HX_{\infty }\frac{\partial \alpha }{\partial t}$$

Here, ρ is the polymer density, u is the velocity in the x direction, $\eta_{s}$ is the viscosity of the polymer, $\tau_{xy}$ is the shear stress of the system, T is the temperature, $c_{p}$ is the heat capacity, $k_{p}$ is the thermal conductivity, α is the relative crystallinity, ΔH is the crystallization enthalpy, and $X_{\infty }$ is the maximum crystallinity.

The term for the total shear stress for the crystallization system $\tau_{xy}$ appears in the momentum Equation (1). Hence, it is necessary to describe the constitutive equation of the system. It should be mentioned that there are many rheological models for crystallizing systems, and the “two suspension model” [18] is a widely used one. One can refer to the review [2] for more details. Zheng and Kennedy [18] proposed this model to account for flow-induced crystallization kinetics. They used an elastic dumbbell and rigid dumbbell model to describe the molecular chain conformation and the orientation evolution for the amorphous and semi-crystalline phases. They considered that free energy is the basis for enhancing crystallization kinetics. The two-suspension model is based on the molecular scale; therefore, it can reflect more information for oriented morphology.

Here, the two suspension model of Zheng and Kennedy [18] is used to describe the crystallizing system. The model assumes that the crystallizing system is a suspension of semi-crystalline entities growing and spreading in a matrix of amorphous material. Thus, the total stress τ can be written as [18]:

(3) $$\tau =\tau_{a}+\tau_{sc}$$

where $\tau_{a}$ and $\tau_{sc}$ are the stresses of the amorphous and semi-crystalline phases, respectively.

The amorphous phase can be treated as elastic dumbbells in Newtonian flow. Here, FENE-P dumbbells are used. The constitutive equation and the stress expression are given as [18]:

(4) $$\lambda_{a}(T)\overset{\nabla }{C}+[\frac{1}{1-trC/b}C-I]=0$$

(5) $$\tau_{a}=nkT\left(\frac{C}{1-trC/b}-I\right)$$

where C is the configuration tensor of the elastic dumbbells, $\overset{\nabla }{C}=DC/Dt-{\left(\nabla u\right)}^{T}\cdot C-C\cdot (\nabla u)$ is the upper-convected derivative of C, b is the nonlinear spring parameter, I is the identity tensor, tr(•) is the trace of the matrix, n is the molecule number density, k is the Boltzmann constant, and $\lambda_{a}(T)$ is the relaxation time of the polymer fluid at the temperature T. The relaxation time $\lambda_{a}(T)$ obeys the Arrhenius equation, namely, [18]:

(6) $$\lambda_{a}(T)=\exp[\frac{E_{a}}{R_{g}}(\frac{1}{T}-\frac{1}{T_{0}})]\lambda_{a,0}$$

where $\lambda_{a,0}$ is the relaxation time at the temperature $T_{0}$, and $E_{a}/R_{g}$ is a constant that can be determined from the experimental data.

The semi-crystalline phase can be treated as the rigid dumbbells in Newtonian flow. The evolution equation of the orientation tensor of the rigid dumbbells 〈RR〉 is [18]:

(7) $$\overset{\nabla }{\langle RR\rangle }=-\frac{1}{\lambda_{sc}(\alpha ,T)}\left(\langle RR\rangle -\frac{I}{m}\right)-\dot{\gamma }:\langle RRRR\rangle$$

and the stress expression is [18]:

(8) $$\tau_{sc}=\frac{\eta_{sc}(\alpha ,T)}{\lambda_{sc}(\alpha ,T)}\left[\langle RR\rangle -\frac{I}{m}+\lambda_{sc}(\alpha ,T)\dot{\gamma }:\langle RRRR\rangle \right]$$

where $\dot{\gamma }$ is the deformation tensor, 〈RRRR〉 is the fourth-order orientation tensor, $\lambda_{sc}(\alpha ,T)$ is the relaxation time of the rigid dumbbells as a function of relative crystallinity and temperature, $\eta_{sc}(\alpha ,T)$ is the viscosity of the semi-crystalline phase that also depends on the relative crystallinity and temperature, and m is the number of dimensions (m=2 is used here). The relationship between the relaxation time of the polymeric flow and the rigid dumbbells is [18]:

(9) $$\frac{\lambda_{sc}(\alpha ,T)}{\lambda_{a}(T)}=\frac{{\left(\alpha /A\right)}^{\beta_{1}}}{{\left(1-\alpha /A\right)}^{\beta }}\;\alpha <A$$

and the relationship between the viscosity of the semi-crystalline phase and amorphous phase is [18]:

(10) $$\frac{\eta_{sc}(\alpha ,T)}{\eta_{a}(T)}=\frac{{\left(\alpha /A\right)}^{\beta_{1}}}{{\left(1-\alpha /A\right)}^{\beta }}\;\alpha <A$$

Here, β, $\beta_{1}$ and A are the empirical constants.

It is noted that the effect of crystallinity on the polymer viscosity are mainly based on suspension theories and empirical equations. Equation (10) is both empirical and derived based on suspensions. For more equations for polymer viscosity, one can refer to the review [2].

Quadratic closure approximation is used to calculate the fourth-order configuration tensor in Equation (8), namely:

(11) $$<RRRR{>}_{ijkl}=<RR{>}_{ij}<RR{>}_{kl}$$

where $<RR{>}_{ij}$ and $<RR{>}_{kl}$ are the components of <RR>, and $<RRRR{>}_{ijkl}$ is the component of 〈RRRR〉. For two-dimensional computation, i,j,k,l take values of 1 and 2; for three-dimensional computation, i,j,k,l take values of 1, 2 and 3.

### 2.2. The Model for Nucleation and Growth of Spherulites and Shish-Kebabs at the Microscopic Level

Polymer crystallization in manufacturing exhibits the property that quiescent crystallization and flow-induced crystallization coexist. It is assumed that the spherulitic structure appears in the quiescent crystallization, while the shish-kebab structure appears in the flow-induced crystallization. The former is induced from a thermal gradient while the latter one is induced by flow.

Eder obtained a series of differential equations that consider the spherulites as growing spheres and the shish-kebabs as growing cylinders [19]. In our previous work [20], we deduced the equivalent differential equations for spherulites and shish-kebabs. It is reported that spherulites are determined by the nucleation density $N_{s}$ and growth rate $G_{s}$, and shish-kebabs are determined by the nucleation density $N_{s-k}$, length growth rate $G_{s-k,l}$ and radius growth rate $G_{s-k,r}$.

Here, nucleation density of the spherulites $N_{s}$ is assumed to obey the following empirical equation [21]:

(12) $$N_{s}(T)=\exp\left\lfloor \tilde{a}(T_{m}^{0}-T)+\tilde{b}\right\rfloor$$

where $\tilde{a}$ and $\tilde{b}$ are the empirical parameters and $T_{m}^{0}$ is the equilibrium melting temperature. Koscher and Fulchiron [21] performed the experiment and used a microscope to determine the relationship between $N_{s}$ and the supercooling $T_{m}^{0}-T$. They used data fitting to obtain the values of $\tilde{a},\tilde{b}$.

The growth rate of the spherulites $G_{s}$ obeys the Hoffman-Lauritzen expression, namely, [22]:

(13) $$G_{s}(T)=G_{0}\exp\left[-\frac{U^{*}}{R_{g}(T-T_{\infty })}\right]\exp\left(-\frac{K_{g}}{T\Delta T}\right)$$

where $U^{*}$ is an energy parameter similar to an apparent activation energy of motion, $R_{g}$ is the gas constant, $T_{\infty }=T_{g}-30$ is considered as the temperature at which no further molecular displacement is possible, and $G_{0}$ and $K_{g}$ are experimentally determined constants. The Hoffman-Lauritzen expression is typically adopted to describe the growth rate. Parameters for different polymers in this expression can be found inform the literature.

The nucleation density of shish-kebabs $N_{s-k}$ is assumed to be a function of the first normal stress difference of the crystallizing system $N_{1}$, which is:

(14) $${\dot{N}}_{s-k}=CN_{1}$$

where C is the constant, $N_{1}=\tau_{xx}-\tau_{yy}$ which is calculated by Equation (3). This equation is proposed by Koscher and Fulchiron [21]. They considered that the flow promoted the enhancement of nucleation and proposed the simplest mathematical relationship between the activated nuclei of shish-kebabs and the first normal stress difference. Although, the nucleation density of shish-kebabs may depends on other variables, i.e., shear rate, pressure, etc., this expression is widely used in numerical simulation works [17,21].

The length growth rate of shish-kebabs $G_{s-k,l}$ can be written as:

(15) $$G_{s-k,l}={\dot{\gamma }}^{2}\cdot g_{l}/{\dot{\gamma }}_{l}^{2}$$

where $g_{l}/{\dot{\gamma }}_{l}^{2}$ is a constant and $\dot{\gamma }$ is the shear rate. Equation (15) is deduced from Eder’s model [3], details of which can be found in our previous work [20].

According to many researchers [3], the radius growth rate of shish-kebabs $G_{s-k,r}$ is assumed to be equal to the growth rate of spherulites $G_{s}$, namely:

(16) $$G_{s-k,r}=G_{s}$$

## 3. Macro-Micro Algorithm

**Figure 3:**
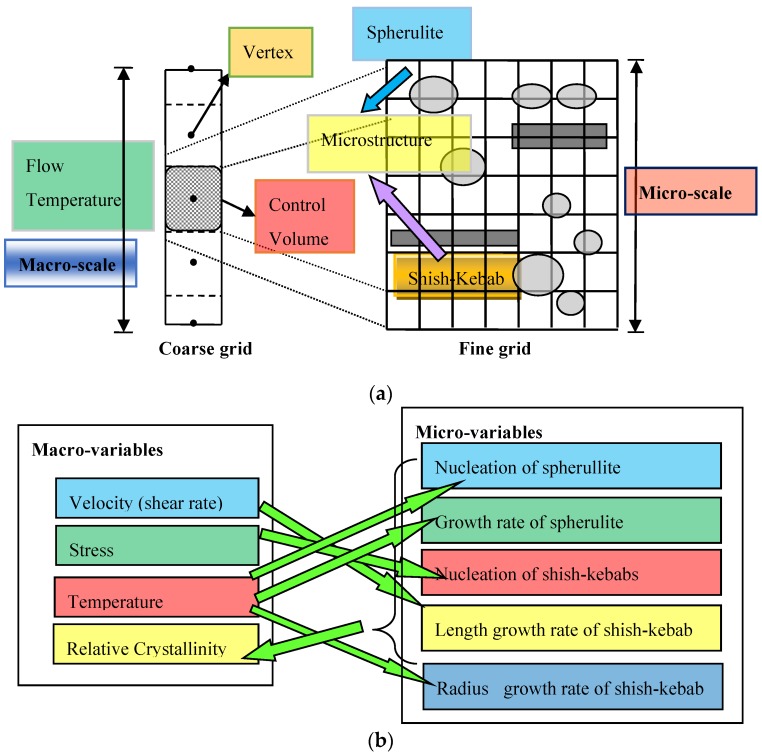
Schematic representation of macro-micro algorithm: (**a**) coarse grid and fine grid; (**b**) the interaction between the macro-variables and the micro-variables in a control volume.

The idea for our macro-micro algorithm was to build up and couple different numerical methods at different scales. Numerical methods at the macroscopic level that simulate the non-isothermal polymeric flow include the finite element method, finite volume method, spectral method, etc. [23]. Numerical methods at the microscopic level that capture the details of crystals include the front tracking method [4], pixel coloring method [10,11], cellular automaton method [7,24], Monte Carlo method [20,25], level set method [8], etc. Here, we constructed a hybrid algorithm of a finite volume method with a Monte Carlo method. The finite volume method is used to calculate the heat and mass transfer of the polymeric flow (Equations (1), (2), (4), and (7)) at the macroscopic level, while the Monte Carlo method is used to capture the development of spherulites (Equations (12) and (13)) and shish-kebabs (Equations (14)–(16)) at the microscopic level. Since the length scale of the crystal morphology and the products varies by several orders of magnitude, we used two different grids to carry out our algorithm, which is shown in Figure 3a. The Couette flow as shown in Figure 2 is a typical one-dimensional flow in *y* direction. Therefore, we assumed that each vertex in the *y* direction has a control volume, which is shown in Figure 3a. The crystal morphology is captured in this two-dimensional square control volume with a height (Δy) determined by macroscopic computations. This is because in Couette flow, the relative crystallinity α appears in the energy Equation (2). To calculate the relative crystallinity, the crystal morphology is needed. In one dimension, the crystal morphologies of spherulites and shish-kebabs are difficult to define, which is not the case in two dimensions. In two dimensions, the spherulite is treated as a growing circle and the shish-kebab is treated as a growing rectangle, which is relatively easy to implement numerically. In addition, the results in two dimensions are more believable than that in one dimension. In our approach, the product is first divided into a coarse grid with the vertex at the center. Each coarse grid (a control volume) is then subdivided into a fine grid. The finite volume method is implemented on the coarse grid to calculate the one-dimensional flow and temperature field at the macroscopic level. The Monte Carlo method is then carried out to capture the two-dimensional crystal morphology on a fine grid. This construction concept for a multi-scale algorithm is similar to our previous works [10,11]. However, our previous works only dealt with the quiescent crystallization, while the present work is more complex and considers the flow-induced crystallization.

Figure 3b schematically shows the interaction between the macro-variables and the micro-variables in a control volume. As seen in Figure 3a, the vertex of the coarse grid is at the center of the control volume. This vertex carries four main pieces of information, namely, the velocity, stress, temperature and relative crystallinity. We assume that these four variables in the control volume are unchanged and equal to the value at the vertex. From the model of nucleation and growth of the spherulites and shish-kebabs at the microscopic level, it is clear that the development of spherulites is determined by the temperature and the development of shish-kebabs is determined by the stress, shear rate and temperature. On the other hand, with the evolution of the crystal morphology at the microscopic level, the macro-variable of the relative crystallinity increases, which may affect other macro-variables, such as temperature, stress and velocity.

### 3.1. Finite Volume Method at the Macroscopic Level

The finite volume method was performed on a coarse grid to calculate the temperature and the flow fields, as shown in Figure 3a. The boundary vertex had a half control volume, while the internal vertex had an entire control volume. However, when we use the Monte Carlo method on the fine grid, the boundary vertex is supposed to include the entire control volume.

The reason that we chose the finite volume method was that the vertex has a definite control volume. The evolution of the crystals in a control volume will directly affect the relative crystallinity at the corresponding vertex. Meanwhile, the changes in temperature and flow field on the vertex will also directly affect the development of the crystals in the control volume.

Equations (1) and (2) are solved using the finite volume method using a uniform grid. Suppose that the computational domain W is divided into N cells. The vertexes are written as $y_{j}=j\cdot \Delta y$ (j=0,1,⋯,N) with Δy=W/N being the step size of the grid. The internal vertex $y_{j}$ has the control volume of $\left[y_{j-1/2},y_{j+1/2}\right]$ (j=1,⋯,N−1).

The forward scheme is used to time discretize and central scheme is used to calculate the flux at the face of the control volume to space discretize. The corresponding equations become:

(17) $$\rho \frac{u_{j}^{n+1}-u_{j}^{n}}{\Delta t}=\eta_{s}\frac{u_{j+1}^{n}-2u_{j}^{n}+u_{j-1}^{n}}{\Delta y^{2}}+\frac{\tau_{xy}{}_{j+1}^{n}-\tau_{xy}{}_{j-1}^{n}}{2\Delta y}$$

(18) $$\rho c_{p}\frac{T_{j}^{n+1}-T_{j}^{n}}{\Delta t}=k_{p}\frac{T_{j+1}^{n}-2T_{j}^{n}+T_{j-1}^{n}}{\Delta y^{2}}+\rho \Delta HX_{\infty }\frac{\alpha_{j}^{n}-\alpha_{j}^{n-1}}{\Delta t}$$

where Δt is the time step, $u_{j}^{n}$ is the velocity at time t=nΔt at vertex $y_{j}$, $T_{j}^{n}$ is the temperature at time t=nΔt at vertex $y_{j}$. It is noted that there is no continuity equation in the governing equations. Therefore, we shall not address the decoupling of pressure and velocity.

The evolution Equations (4) and (7), which represent the configuration tensor C in the amorphous phase and orientation tensor 〈RR〉 in the semi-crystalline phase, respectively, are also solved by the finite volume method. The forward-time scheme is used, and the discrete equations are:

(19) $$\frac{C_{j}^{n+1}-C_{j}^{n}}{\Delta t}=-\frac{1}{\lambda_{a}(T_{j})}[\frac{1}{1-trC_{j}^{n}/b}C_{j}^{n}-I]+{\left(\nabla u\right)}_{j}^{T,n}\cdot C_{j}^{n}+C_{j}^{n}\cdot {\left(\nabla u\right)}_{j}^{n}$$

(20) $$\frac{{\langle RR\rangle }_{j}^{n+1}-{\langle RR\rangle }_{j}^{n}}{\Delta t}=-\frac{1}{\lambda_{sc}(\alpha_{j},T_{j})}\left({\langle RR\rangle }_{j}^{n}-\frac{I}{m}\right)-{\dot{\gamma }}_{j}:{\langle RRRR\rangle }_{j}^{n}+{\left(\nabla u\right)}_{j}^{T,n}\cdot {\langle RR\rangle }_{j}^{n}+{\langle RR\rangle }_{j}^{n}\cdot {\left(\nabla u\right)}_{j}^{n}$$

Here, $\nabla u_{j}^{n}\approx \left(\begin{array}{cc} 0 & \left(u_{j+1}^{n}-u_{j-1}^{n})/(2\Delta y\right)\\ 0 & 0 \end{array}\right)(j=1,\cdots ,N-1)$, $\nabla u_{0}^{n}\approx \left(\begin{array}{cc} 0 & (u_{1}^{n}-u_{0}^{n})/\Delta y\\ 0 & 0 \end{array}\right)$, $\nabla u_{N}^{n}\approx \left(\begin{array}{cc} 0 & (u_{N}^{n}-u_{N-1}^{n})/\Delta y\\ 0 & 0 \end{array}\right)$.

When the configuration tensor C and orientation tensor 〈RR〉 have been calculated, the stress of the amorphous phase $\tau_{a}$ and the semi-crystalline phase $\tau_{sc}$ can be calculated with Equations (5) and (8), respectively. The sum of these two stresses affects the right source term in Equation (17).

### 3.2. The Model for Heat and Mass Transfer of Polymeric Flow at the Macroscopic Level

The Monte Carlo method is implemented on a fine grid at the microscopic level. The main advantages of the Monte Carlo method are that the crystal morphology evolution can be obtained, and the relative crystallinity can be calculated statistically from the crystal morphology evolution. This avoids using the crystallization kinetics model.

In our previous work, a parametric study of flow-induced crystallization [25] and polymer crystallization in a 3D simple shear flow [20] have shown the detailed implementation of the Monte Carlo method to capture the growth front of both spherulites and shish-kebabs. Here, we stress that the relative crystallinity is calculated by the volume fraction of the crystals by:

(21) α=number of cells that are occupied by crystals/total number of cells

The details of the Monte Carlo method can be found in our previous works [20,25].

### 3.3. Implement of the Macro-Micro Algorithm

The macro-micro algorithm is implemented as follows.

**STEP 1**: Initialization. At time t=0 s, set the internal velocity $u_{j}^{0}=0$ (j=1,⋯,N−1), the internal temperature $T^{0}=T_{0}$ (j=1,⋯,N−1), internal configuration tensor $C_{j}^{0}=I/2$ (j=1,⋯,N−1), internal orientation tensor ${\langle RR\rangle }_{j}^{0}=I/2$ (j=1,⋯,N−1), and the relative crystallinity $\alpha_{j}^{0}=0$ (j=0,⋯,N).

**STEP 2**: Solve the flow field (Equation (17)) and temperature (Equation (18)) equations using the finite volume method with the boundary conditions $u_{0},u_{N},T_{0},T_{N}$. Solve the evolution equations of the configuration tensor C in the amorphous phase and orientation tensor 〈RR〉 in the semi-crystalline phase (Equations (19) and (20)) using the finite volume method, and calculate the stresses with Equations (5) and (8).

**STEP 3**: Capture the growth fronts of the spherulites and shish-kebabs with the Monte Carlo method and compute the relative crystallinity using the volume fraction of the crystals. With the parameters obtained from STEP 2, calculate the nucleation density and growth rate of the spherulites with Equations (12) and (13), and the nucleation density, length growth rate, and radius growth rate of the shish-kebabs with Equations (14)–(16). Use the Monte Carlo method [20,25] to capture the development of the crystals and use Equation (21) to calculate the relative crystallinity.

**STEP 4**: Go to **STEP 2** until the time has reached $t_{end}$.

## 4. Results and Discussion

The polymer used in this work is polypropylene. The material parameters are listed as: momentum and energy equations are [10] $\rho =900\;\mathrm{Kg}\cdot m^{-3}$, $c_{p}=2.14\times 10^{3}\;J\cdot {\mathrm{Kg}}^{-1}\cdot K^{-1}$, $k_{p}=0.193\;W\cdot m^{-1}\cdot K^{-1}$, and $\Delta HX_{\infty }=107\times 10^{3}\;J\cdot \mathrm{Kg}$; nucleation and growth rates are [21] $\tilde{a}=1.56\times 10^{-1}\;K^{-1}\cdot m^{-3}$, $\tilde{b}=1.51\times 10^{1}\;m^{-3}$, $G_{0}=2.83\times 10^{2}\;m\cdot s^{-1}$, $U^{*}/R_{g}=755\;K$, $K_{g}=5.5\times 10^{5}\;K^{2}$, $T_{m}^{0}=483\;K$, and $T_{g}=269\;K$, shish-kebab parameter are [21] $g_{l}/{\dot{\gamma }}_{l}{}^{2}=2.69\times 10^{-7}$, and $C=10^{6}\;{\mathrm{Pa}}^{-1}\cdot s^{-1}\cdot m^{-1}$, parameters for the two-suspension model are [18] $\lambda_{a,0}=4.00\times 10^{-2}\;s$, $T_{0}=476.15\;K$, $E_{a}/R_{g}=5.602\times 10^{3}\;K$, b=5, $n=1.26\times 10^{26}/m^{3}$, $k=1.38\times 10^{-23}$, β=9.2, $\beta_{1}=0.05$, and A=0.44.

### 4.1. Validity of Model and Algorithm on the Single-Scale

#### 4.1.1. Validity of Finite Volume Method at the Macroscopic Level

**Figure 4:**
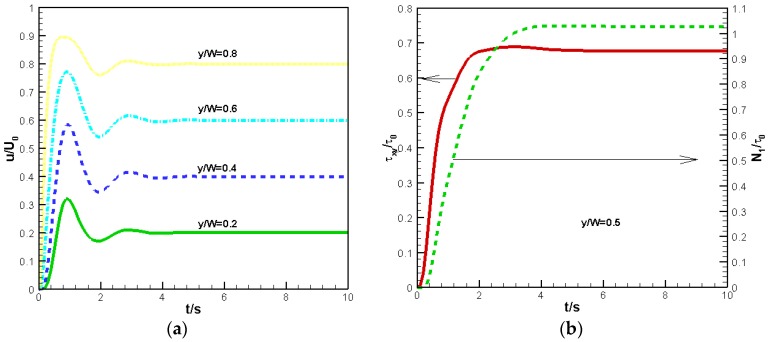
Evolution of velocity and stress: (**a**) velocity (**b**) shear stress and first normal stress difference.

To verify the accuracy of the finite volume method used at the macroscopic level, we consider Couette flow under isothermal conditions. As shown in Figure 2, the upper wall is moved to the right with a velocity $u=\dot{\gamma }W$ at t=0 s and the temperature is set to T=100 °C. Here, we assume $\dot{\gamma }=1\;s^{-1},W=1\;\mathrm{mm}$. In our simulation, N=10 cells are used in the y direction. To compare with other numerical results, we neglect the effects of the semi-crystalline phase on the momentum equation and only consider Equations (1), (4), and (5). Figure 4a,b shows the velocity evolution at different locations and the shear stress evolution $\tau_{xy}$ and first normal stress difference $N_{1}$ at the midpoint. In Figure 4, the velocity and stress are reduced by $U_{0}=\dot{\gamma }W$ and $\tau_{0}=\eta_{0}U_{0}/W$, respectively. Their evolution tendency and the numerical data are in good agreement with the numerical results of Ouyang et al. [26].

#### 4.1.2. Validity of Model and Monte Carlo Method at the Microscopic Level

**Figure 5:**
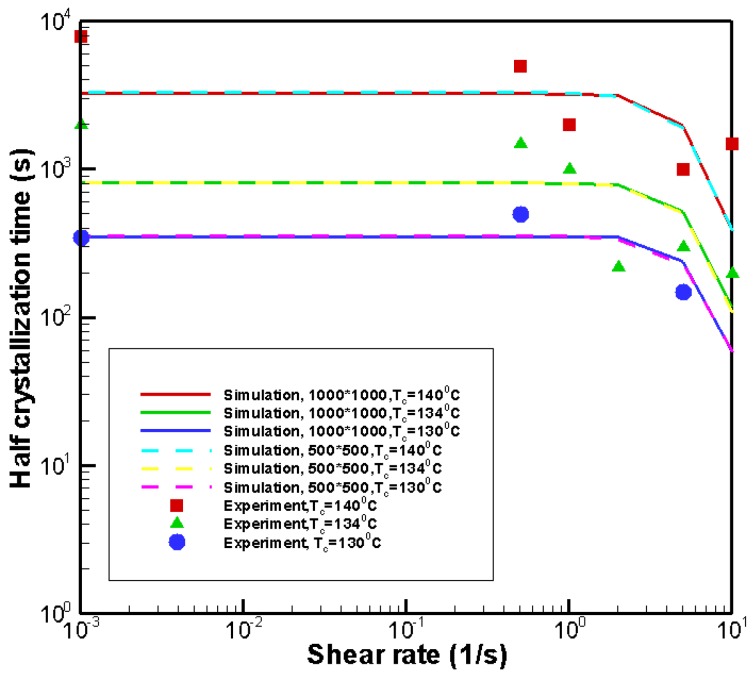
Comparison of simulation result with the experimental result [21].

**Figure 6:**
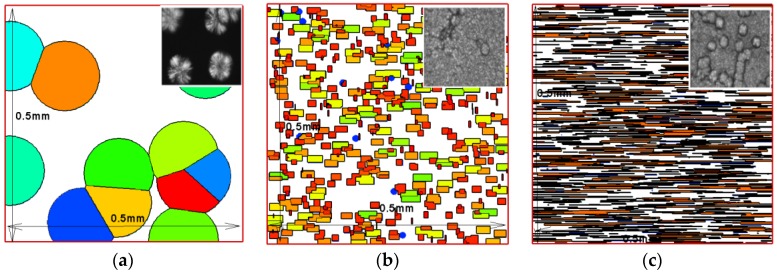
Comparison of simulated crystal morphology after shear treatments with the experimental result [21]: (**a**) without shear; (**b**) $\dot{\gamma }=5\;s^{-1},t_{s}=10\;s$; (**c**) $\dot{\gamma }=5\;s^{-1},t_{s}=30\;s$.

To verify the effectiveness of the model and the Monte Carlo method used at the microscopic level, we consider isothermal crystallization. Here, the Monte Carlo method is implemented over a small region, 0.5 mm×0.5 mm. This small region is divided into a large number of squares (fine grid), 1000×1000 and 500×500. Figure 5 compares the numerical results with the experimental data from Koscher and Fulchiron [21] at the shear time of $t_{s}=10\;s$. The Monte Carlo method is implemented five times to calculate the average of half crystallization times. The numerical relationship between the shear rate and the half crystallization time was in good agreement with the experimental data. Thus, the model and the Monte Carlo method used at the microscopic level was valid to predict the crystallization kinetics. In addition, there were slight differences in the Monte Carlo results obtained with the grid numbers of 1000×1000 and 500×500. Therefore, it can be concluded that the result obtained with the grid numbers of 500×500 are reliable. Figure 6 shows the simulated crystal morphology at the crystallization temperature $T_{c}=140\;{}^{\circ}C$ with different shear treatments for the 500×500 grid. The tendency of the crystal morphology is consistent with Koscher and Fulchiron’s experimental results [21]. Therefore, it can be concluded that the model and the Monte Carlo method used at the microscopic level can also predict the correct trends in the crystal morphology evolution.

### 4.2. Numerical Results Obtained by the Macro-Micro Model and Algorithm

Considering the Couette flow shown in Figure 2, the upper wall moves to the right at a velocity $u=\dot{\gamma }W$ at t=0 s while the lower wall remains stationary. When the time reaches the shear time $t_{s}$, the upper wall stops moving. The temperature of the lower wall is set to $T_{w}$ while the temperature of the upper wall is set to ∂T/∂y=0. These boundary conditions are related to polymer manufacturing processing. Here, the lower wall is considered as the mold wall, while the upper wall is considered as the core layer of the polymer. We should mention that the numerical example we considered here is the same as Zinet et al. [15]. In our simulation, the cell number of the coarse grid is N=4, and the cell numbers of fine grid are 500×500. The initial temperature is set to the equilibrium melting temperature $T_{m}^{0}$. Unless otherwise stated, the parameters in the simulation are W=2 mm, $\dot{\gamma }=10\;s^{-1}$, $t_{s}=5\;s$, $T_{w}=60\;{}^{\circ}C$, Δy=0.5 mm, and Δt=0.01 s.

#### 4.2.1. Macro-Micro Results

**Figure 7:**
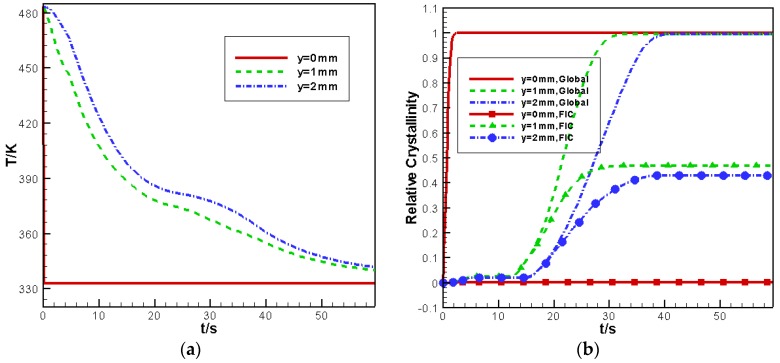
Temperature and relative crystallinity evolution at different *y* locations: (**a**) temperature (**b**) relative crystallinity.

Figure 7 shows the evolution of temperature and relative crystallinity with time at different y locations. As seen in Figure 7, the lowest temperature at the lower wall (y=0 mm) leads to the quickest crystallization. In addition, due to the cooling effect of the lower wall, the temperature cooling rate at y=1 mm is higher than at y=2 mm. Therefore, the crystallization rate at y=1 mm is higher than that y=2 mm. In addition, as shown in Figure 7a, the temperature has produced a “platform” at the core layer (y=2 mm) at approximately t=20~38 s. With the help of Figure 7b, it is easy to find that this “platform” is caused by latent heat released by crystallization. Figure 7b also shows that the relative crystallinity contributed by flow-induced crystallization (FIC) at different y locations. When the crystallization is completed, the contribution of FIC at the lower wall y=0 mm is 0%; at the middle plane y=1 mm is 47%; and at the upper wall y=2 mm is 43%. This trend is consistent with the numerical results of Zinet et al. [15].

It is worth noting that the final results are sensitive to the time steps and cell numbers of the coarse grid. In fact, Δt,Δy appear in the finite volume scheme of Equations (17) and (18). The errors in these schemes are on the order of $O(\Delta t)+O(\Delta y^{2})$. It is apparent that the smaller time steps and the larger cell numbers of the coarse grid lead to more accurate results at the macroscopic level. However, the cell numbers of the coarse grid should not be too large. When the cell numbers of coarse grid increases, the length of the control volume (Δy) decreases. Thus, the nucleation number of the spherulites and shish-kebabs in one control volume is smaller. To calculate the reliable temperature, flow field and relative crystallinity, the Monte Carlo method should be implemented more times to determine the average. Therefore, the large numbers for the coarse grid or the small size of Δy not only affect the computation cost, but also affect the accuracy of the results.

**Figure 8:**
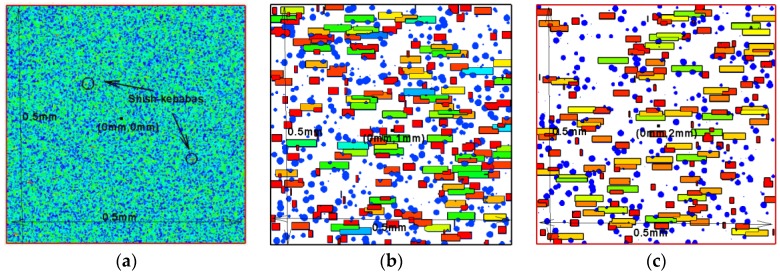
Crystal evolution in different control volume: (**a**) y=0 mm; (**b**) y=1 mm; (**c**) y=2 mm.

The macro-micro simulation allowed us to see the detailed microstructure. Figure 8 shows the evolution of the crystal morphology in the control volume at different y locations. In the control volume at y=0 mm, the lowest temperature promises the largest nucleation density of spherulites. In addition, since the crystallization finishes relatively quickly, the shear effect is at weaker proximity to this wall. Therefore, in the control volume at y=0 mm, shish-kebab structures were not so obvious. From the crystal morphology evolution in the control volume at y=1 mm and y=2 mm, it was apparent that shish-kebabs were produced due to the shear effect. Therefore, in these control volumes, both spherulites and shish-kebabs contributed to the relative crystallinity.

Note that the crystal morphology at the microscopic level was sensitive to the fine grid cell numbers. Generally, the larger the cell numbers, the more accurate the crystal morphology and relative crystallinity are. However, the larger fine grid cell numbers lead to greater computational cost. Therefore, one may balance the accuracy and computational cost to choose suitable cell numbers for the fine grid.

#### 4.2.2. Effects of Shear Rate

**Figure 9:**
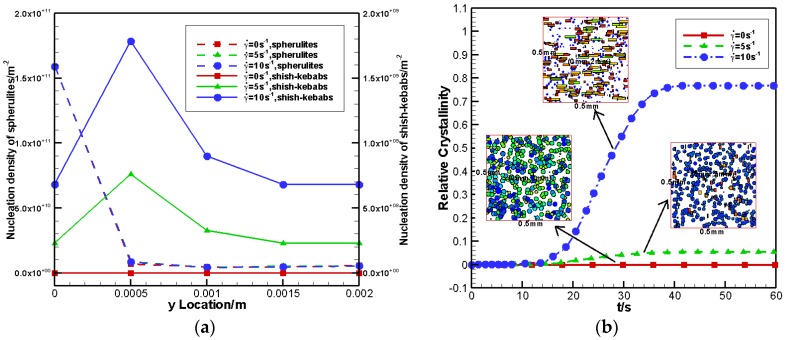
Effects of shear rate on nucleation density and relative crystallinity: (**a**) nucleation density of spherulites and shish-kebabs at different y locations (**b**) relative crystallinity caused by flow-induced crystallization (FIC) and crystal morphology with different shear rates.

Here, three shear rates were considered, namely, $\dot{\gamma }=0\;s^{-1},5\;s^{-1},10\;s^{-1}$. Shear time was kept constant at $t_{s}=5\;s$. Figure 9a shows the nucleation density of the spherulites and shish-kebabs at different y locations. It is clear that the increase in the shear rate significantly increased the nucleation density of shish-kebabs. However, its effect on the nucleation density of spherulites was very small. To study the effects of shear rate on the relative crystallinity and crystal morphology, the control volumes at y=2 mm (core layer) were taken as an example. Figure 9b plots the relative crystallinity caused by the FIC and crystal morphology with different shear rates. As shown in Figure 9b, the higher the shear rate, the larger the contribution and the higher anisotropy of flow-induced shish-kebabs. These results were similar to our previous work [20] where 3D simple shear flow was considered.

#### 4.2.3. Effects of Shear Time

**Figure 10:**
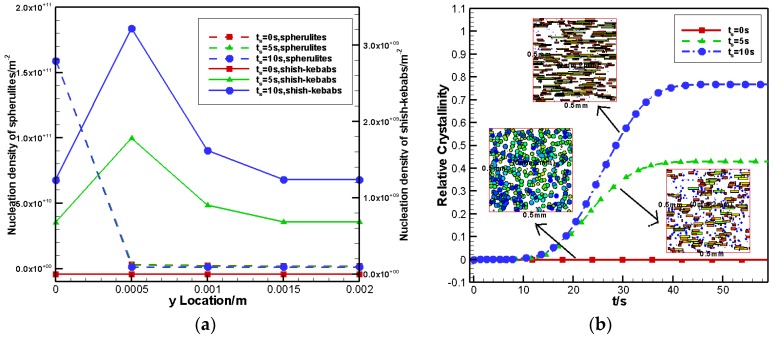
Effects of shear time on nucleation density and relative crystallinity: (**a**) nucleation density of spherulites and shish-kebabs at different y locations (**b**) relative crystallinity caused by FIC and crystal morphology with different shear time.

Here, three cases of shear time are analyzed, namely, $t_{s}=0\;s,5\;s,10\;s$, and the shear rate was set to $\dot{\gamma }=10\;s^{-1}$. Figure 10a shows the nucleation density of spherulites and shish-kebabs at different y locations. The increased shear time mainly affected the nucleation density of the shish-kebabs by increasing their density. Figure 10b displays the contribution of the FIC on the relative crystallinity and the crystal morphology in the control volume at y=2 mm (core layer) with different shear times. It can be seen from Figure 10b that the longer the shear time, the bigger the contribution and the higher the anisotropy of the flow-induced shish-kebabs. This result was also consistent with the experimental results of Kornfield et al. [27] and our previous work of 3D simple shear flow [20].

#### 4.2.4. Effects of Wall Temperature

**Figure 11:**
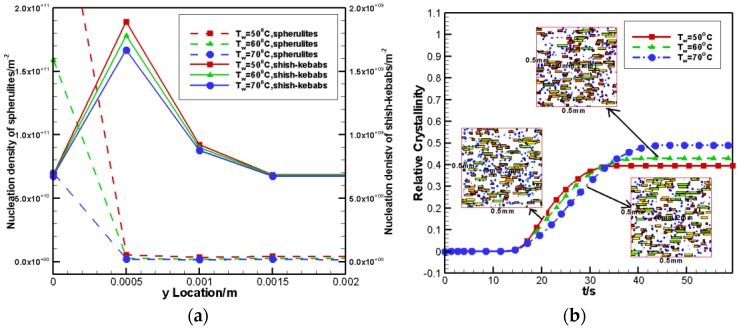
Effects of wall temperature on nucleation density and relative crystallinity: (**a**) nucleation density of spherulites and shish-kebabs at different y locations (**b**) relative crystallinity caused by FIC and crystal morphology with different wall temperature.

Wall temperature is also an important factor for crystallization. Here, three cases of wall temperature were considered, namely, $T_{w}=50\;{}^{\circ}C,60\;{}^{\circ}C,70\;{}^{\circ}C$. The shear rate was set to $\dot{\gamma }=10\;s^{-1}$ and the shear time is set to $t_{s}=5\;s$. Figure 11a gives the nucleation density of the spherulites and shish-kebabs at different y locations. Due to the change in wall temperature, the nucleation density of spherulites changes greatly. A lower wall temperature leads to a higher cooling rate in the internal product, thus a higher nucleation density of spherulites is observed. In contrast, the change of wall temperature has little effect on the nucleation density of the shish-kebabs. Figure 11b plots the relative crystallinity as contributed by the FIC and the crystal morphology at the core layer of the control volume at y=2 mm. It can be found that the lower the wall temperature, the smaller the contribution of flow-induced shish-kebabs is. Since the relative crystallinity is caused by both spherulites and shish-kebabs, it is also apparent that the lower the temperature, the larger the contribution of spherulites is.

## 5. Conclusions

We establish a macro-micro model and a macro-micro algorithm for the polymer crystallization in Couette flow. Our model and algorithm can handle both quiescent crystallization and flow-induced crystallization. They can also predict the temperature, relative crystallinity at the macroscopic level and capture the crystal morphology development at the microscopic level. Based on the macro-micro model and macro-micro algorithm, we explored the effects of shear rate, shear time, and wall temperature on the crystal morphology and relative crystallinity. Conclusions are drawn as follows:

(1) By comparing our simulated results with the experimental data and other numerical results, we can conclude that the macro-micro model and the macro-micro algorithm were effectively constructed. Our simulated results not only predicted the change in temperature and relative crystallinity, but also predicted the correct trends in the spherulite and shish-kebab evolution.
(2) The increase of the shear rate or shear time increased the contribution of shish-kebabs on the relative crystallinity, and caused high nucleation density and high anisotropy of shish-kebabs.
(3) The decrease of the wall temperature increased the contribution of spherulites to the relative crystallinity, and causes a high nucleation density of spherulites.

This paper presents the concepts of a macro-micro model and macro-micro algorithm for polymer crystallization. The macro-micro model and macro-micro algorithm are not limited. For example, other rheological models can be used in the macro model; other nucleation and crystal growth models can be used in the micro model; FEM, FDM, etc. can be used in the macro method; and the cellular automaton method, level set method, etc. can be used in the micro method. We hope the idea of this multi-scale model and multi-scale method will give more insight into the simulation of polymer crystallization.
